# A haven for an extremely disturbed young person

**DOI:** 10.1192/bjo.2021.340

**Published:** 2021-06-18

**Authors:** Rummana Khan

**Affiliations:** Consultant Child & Adolescent Psychiatrist, Leicester Partnership NHS Trust

## Abstract

**Objective:**

The reason to share this case is to high light the lack of resources in mental health services which can delay the provision of appropriate care and this can have negative impact on child health outcomes.

**Background:**

A 10-year-old boy was referred to CAMHS. He presented with extremely challenging behaviours. After first appointment with CAMHS he attacked his father and nurses. He had to be restrained multiple times. He started to use wooden chair as a weapon, threatened to harm others and threatened to urinate on staff. He tried to kill him-self by ligature. Mental health act assessment was completed and when a decision was reached that detention under the mental health act was appropriate, no appropriate bed was available. He was admitted under Section II of MHA to paediatric ward where he remained for one week (with 2:1 CAMHS support). Then he was transferred to an inpatient CAMHS unit which was commissioned for children over 12 years of age. At a later date mental health tribunal panel upheld the section. After few days he was transferred to an age appropriate in-patient mental health bed. He stayed there for roughly 6 months and was discharged with a diagnosis of ADHD and Autistic Spectrum Disorder. There was a long delay in discharge, until appropriate specialist residential placement could be identified and he was transferred there. He is well settled now in the placement.

**Case report:**

Legal advice was later taken on this case. MHA 1983, Human Rights Act, Children Act 1989, Criminal Law Act 1967 and Code of Practice 2015 were considered and it was agreed that it was appropriate to use MHA 1983. There was discussion whether the Children Act could be relied on instead, but in view of the fact that repeated restrain was required, he was in seclusion and possibility of need for rapid tranquilization post admission the decision was made to use the mental health act.

**Conclusion:**

This case has highlighted a significant problem and calls for an urgent action to increase the number of inpatient age appropriate mental health beds and number of appropriate residential placements nationally. It has also been identified that application of legal frame work in children and adolescents can be a challenge and there is a need for targeted educational programmes for professionals on the use of legal frame work in children and adolescents.

